# *Euphorbia cuneata* Represses LPS-Induced Acute Lung Injury in Mice via Its Antioxidative and Anti-Inflammatory Activities

**DOI:** 10.3390/plants9111620

**Published:** 2020-11-21

**Authors:** Hossam M. Abdallah, Dina S. El-Agamy, Sabrin R. M. Ibrahim, Gamal A. Mohamed, Wael M. Elsaed, Amjad A. Elghamdi, Martin K. Safo, Azizah M. Malebari

**Affiliations:** 1 Department of Natural Products and Alternative Medicine, Faculty of Pharmacy, King Abdulaziz University, Jeddah 21589, Saudi Arabia; gahussein@kau.edu.sa (G.A.M.); aalghamdi2861@stu.kau.edu.sa (A.A.E.); 2 Department of Pharmacognosy, Faculty of Pharmacy, Cairo University, Cairo 11562, Egypt; 3 Department of Pharmacology and Toxicology, Faculty of Pharmacy, Mansoura University, Mansoura 35516, Egypt; dagamiabdalla@taibahu.edu.sa; 4 Department of Pharmacology and Toxicology, College of Pharmacy, Taibah University, University Street, Al-Madinah Al-Munawarah 30078, Saudi Arabia; 5 Department of Pharmacognosy, Faculty of Pharmacy, Assiut University, Assiut 71526, Egypt; sabreen.ibrahim@pharm.au.edu.eg; 6 Department of Pharmacognosy, Faculty of Pharmacy, Al-Azhar University, Assiut Branch, Assiut 71524, Egypt; 7 Department of Anatomy and Embryology, Faculty of Medicine, Mansoura University, Mansoura 35516, Egypt; wzaarina@taibahu.edu.sa; 8 Department of Anatomy and Embryology, College of Medicine, Taibah University, University Street, Al-Madinah Al-Munawarah 30078, Saudi Arabia; 9 Department of Medicinal Chemistry, Institute for Structural Biology, Drug Discovery and Development, School of Pharmacy, Virginia Commonwealth University, Richmond, VA 23219, USA; msafo@vcu.edu; 10 Department of Pharmaceutical Chemistry, Faculty of Pharmacy, King Abdulaziz University, Jeddah 21589, Saudi Arabia; amelibary@kau.edu.sa

**Keywords:** *Euphorbia cuneata*, Euphorbiaceae, lipopolysaccharide, oxidative stress, NF-κB, lung injury

## Abstract

*Euphorbia cuneata* (EC; Euphorbiaceae), which widely grows in Saudi Arabia and Yemen, is used traditionally to treat pain and inflammation. This study aimed to evaluate the protective anti-inflammatory effect of a standardized extract of EC against lipopolysaccharide (LPS)-induced acute lung injury (ALI) in mice and the possible underlying mechanism(s) of this pharmacologic activity. ALI was induced in male Balb/c mice using intraperitoneal injection of LPS. A standardized total methanol extract of EC or dexamethasone was administered 5 days prior to LPS challenge. Bronchoalveolar fluid (BALF) and lung samples were collected for analysis. The results demonstrated the protective anti-inflammatory effect of EC against LPS-induced ALI in mice. Standardized EC contained 2*R*-naringenin-7-*O*-*β*-glucoside (**1**), kaempferol-7-*O*-*β*-glucoside (**2**), cuneatannin (**3**), quercetin (**4**), and 2*R*-naringenin (**5**) in concentrations of 6.16, 4.80, 51.05, 13.20, and 50.00 mg/g of extract, respectively. EC showed a protective effect against LPS-induced pulmonary damage. EC reduced lung wet/dry weight (W/D) ratio and total protein content in BALF, indicating attenuation of the pulmonary edema. Total and differential cell counts were decreased in EC-treated animals. Histopathological examination confirmed the protective effect of EC, as indicated by an amelioration of LPS-induced lesions in lung tissue. EC also showed a potent anti-oxidative property as it decreased lipid peroxidation and increased the antioxidants in lung tissue. Finally, the anti-inflammatory activity of EC was obvious through its ability to suppress the activation of nuclear factor-κB (NF-κB), and hence its reduction of the levels of downstream inflammatory mediators. In conclusion, these results demonstrate the protective effects of EC against LPS-induced lung injury in mice, which may be due to its antioxidative and anti-inflammatory activities.

## 1. Introduction

Acute lung injury (ALI) associated with sepsis is a common clinical problem with a high morbidity rate [1,2]. The pathogenesis of ALI involves disruption of epithelial integrity with massive infiltration of inflammatory cells into the lung tissue, leading to pulmonary edema and severe inflammation [3,4]. Infiltered inflammatory cells, mainly neutrophils and macrophages, release inflammatory mediators such as interleukins (ILs), tumor necrosis factor (TNF)-α, and nitric oxide (NO) [5,6]. Nuclear factor-κB (NF-κB) is a pro-inflammatory transcription factor that regulates and controls inflammatory response during ALI. NF-κB is present in the cytosol in inactive state due to linkage to its inhibitory protein (IκB). Upon stimulation, IκB rapidly degrades to liberate NF-κB, which then migrates into the nucleus where it potentiates gene expression of inflammatory mediators, hence inducing inflammatory responses [7,8].

Lipopolysaccharide (LPS) is bio-active component of the Gram-negative bacterial cell wall [9], and has been used to establish a mouse model of ALI [5]. LPS activates reactive oxygen species (ROS) generation and the release of proteases. Furthermore, LPS induces the activation of NF-κB signaling pathway and cytokine release [10,11]. Inflammatory mediators activate overproduction of ROS, leading to more oxidative damage. Therefore, suppression of oxidative stress and/or inflammation is a potential strategy to improve ALI.

Many people rely on traditional medicine for their primary healthcare, and it is increasingly becoming popular throughout the world. Medicinal plants have also attracted much attention by researchers that are looking for new leads for developing drugs to treat various ailments [12]. Euphorbiaceae is a large plant family comprising about 320 genera with 7950 species, which are distributed mainly in temperate and tropical regions. Some species of this family are of medicinal and economic importance [13]. *Euphorbia* is one of the largest genera of this family, containing approximately 2160 plant species, which are characterized by milky irritant latex [13,14]. Several plants of this genus are used to treat diarrhea, dysentery, gonorrhea, gastric disorders, edema, warts, whooping cough, asthma, and migraine [14,15,16]. Some of these plants have also been shown to have spasmolytic, diuretic, anti-inflammatory, analgesic, antileukemic, wound healing, hemostatic, and anti-hemorrhoid activities [13,15,16,17]. This genus is known to possess several phytochemicals, e.g., tannins, phenolic compounds, terpenoids, and flavonoids. *Euphorbia cuneata* Vahl. (EC), one of the plant species in this genus, has various traditional uses in Saudi Arabia and Yemen [15,18,19]. The stem juice when mixed with water or milk is used to treat obesity, food poisoning, and constipation [18]. Moreover, in parts of northern Yemen, the stem juice is used on wound and injuries to reduce external bleeding. It is also used to treat postpartum hemorrhage, and also reported to have analgesic and anti-inflammatory activities [15,18,19]. *E. cuneata* was recently characterized as containing several phytochemicals, including triterpenes and flavonoids [20]. The hemostatic activity of the plant juice is attributed to its flavonoid constituents [15,18]. Unlike several members of the Euphorbiaceae family, *E. cuneata* does not contain the usual toxic diterpenes [21]. On the basis of the traditional anti-inflammatory activity of EC, this study aimed to investigate the possible protective effect of EC against LPS-induced ALI in mice and the possible underlying mechanisms. 

## 2. Materials and Methods

### 2.1. Chemicals

Acetonitrile, methanol, and formic acid (LC-MS-grade) were obtained from J. T. Baker (Avantor Performance Materials, Radnor, PA, USA). Milli-Q water (Merck Millipore Corporation, Billerica, MA, USA) was used for liquid chromatography analysis. 2*R*-Naringenin, quercetin, cuneatannin, 2*R*-naringenin-7-*O*-*β*-glucoside, and kaempferol-7-*O*-*β*-glucoside were isolated previously from *E. cuneata* [20]. Lipopolysaccharide (LPS; *Escherichia coli* serotype O111:B4) was bought from Sigma-Aldrich (St. Louis, MO, USA) and freshly prepared in normal saline on the challenge day. Ketamine was obtained as ampoules (Tekam, Hikma Pharmaceuticals, Amman, Jordan). Dexamethasone was obtained as ampoules (4 mg/mL, EIPICO, Cairo, Egypt). Other chemicals and reagents were of highest purity. Pierce bicinchoninic acid (BCA) Protein Assay Kit was purchased from Thermofisher Scientific (Waltham, MA, USA). Lactate dehydrogenase (LDH) activity kit was purchased from Human (Wiesbaden, Germany). Malondialdehyde (MDA), catalase, superoxide dismutase (SOD), reduced glutathione (GSH), and total antioxidant capacity (TAC) kits were purchased from Bio-diagnostic Co. (Giza, Egypt). The 4-hydroxynonenal (4-HNE) kit was purchased from My BioSource (San Diego, CA, USA). ELISA kits for NF-κB (ab176648) were purchased form Abcam (Cambridge, MA, USA), while tumor necrosis factor-α (TNF-α, MTA00B), interleukin-1β (IL-1β, MLB00C), and IL-6 (M6000B) were purchased from R&D Systems (Minneapolis, MN, USA).

### 2.2. Plant Material

*E. cuneata* aerial parts were collected in March 2016 from Al-Taif City, Saudi Arabia. The plant was kindly identified by a taxonomist at the Department of Natural products and Alternative Medicine, King Abdulaziz University, Saudi Arabia, in addition to its morphological features and the library database [22]. It was confirmed by Dr. Emad Al-Sharif, Associate Professor of Plant Ecology, Department of Biology, Faculty of Science and Arts, Khulais, King Abdulaziz University, Saudi Arabia. A voucher specimen (EC-1036) was archived at the Department of Natural Products and Alternative Medicine herbarium, King Abdulaziz University, Saudi Arabia. 

### 2.3. Extraction Procedures for Pharmacological Study 

The air-dried powdered aerial parts of EC (100 g) were extracted with methanol (2 × 500 mL) using an IKA Ultra-Turrax T 25 digital instrument (IKA Labortechnik, Staufen, Germany). The solvent was removed under reduced pressure and the dried total methanolic extract (TEC) (10.9 g) was kept at 4 °C until use in biological tests.

### 2.4. Extraction Procedures of Plant Material for High Performance Liquid Chromatography Diode-Array Detection

The air-dried powdered aerial parts of EC (1 g) were extracted with methanol (10 mL) as described above. The extract was then vortexed vigorously and centrifuged to remove plant debris. Supernatant was evaporated, and 20 mg of the dry residue (100 mg) was placed on a C18 cartridge preconditioned with methanol and water. The sample was eluted using 3 mL MeOH (100%) and the eluate evaporated. The dry residue was re-suspended in 500 μL methanol, and 3 microliters of the supernatant were used for HPLC analysis.

### 2.5. HPLC Photodiode Array Determination of Flavonoid Content in TEC

The HPLC system consists of an Agilent 1260 system, solvent delivery module, quaternary pump, autosampler, column compartment, and diode array detector (Agilent Technologies, Germany). The control of the HPLC system and data processing were performed using ChemStation (Rev. B.01.03 SR2 (204)). The separation was performed on Kromasil 100 C18, 5 μm, 250 × 4.6 mm column (Teknokroma, S. Coop. C. Ltd., Barcelona, Spain), maintained at 25 ± 2 °C. The LC system was programmed to deliver the mobile system as follows: Diode array detector (DAD): 284, 330, and 360 nm; **mobile A**: 0.2% formic acid in water; **mobile B**: acetonitrile; gradient elution program: 0–10 min, 5% B; 10–60 min, 5%–38% B; 60–61 min, 38%–100% B; 61–65 min, 100% B; 65–66 min, 5% B; run time, 72 min; flow rate, 0.9 mL/min.

### 2.6. Calibration Curve of the Isolated Compounds

A weight of 10 mg of each isolated phenolic compound was transferred to a volumetric flask and dissolved in 10 mL methanol. Serial dilutions were prepared in methanol to achieve concentrations of 25, 50, 100, 150, 200, and 250 ng/μL. Each calibration level was analyzed in triplicate. Each compound was injected separately by applying scan mode DAD from 190–500 nm. The UV-VIS scan of each compound was saved and matched with the detected compounds in each sample.

### 2.7. Biological Study

#### 2.7.1. Animals and Experimental Model 

Male Balb/c albino mice (20–25 g) were held in standard conditions in the Animal Facility, College of Pharmacy, King Abdulaziz University, and provided with standard laboratory food and water. All study procedures were approved by the Research Ethical Committee of King Abdulaziz University, Saudi Arabia (reference number PH-116-40), which follows the National Institutes of Health (NIH) guidelines. Mice were divided into 5 groups (*n* = 8/each group) and were treated as follows: ***control group***: mice were given the vehicle once daily for 5 days; ***LPS group***: to induce acute lung injury, we injected LPS (10 mg/kg) intraperitoneally, as previously described [4]; ***EC + LPS groups***: 2 animal groups that were orally administered EC at 2 different dose levels (25 and 50 mg/kg) for 5 days prior to LPS injection; dexamethasone **(*DEX*) *+ LPS* group**: a positive control group where mice were administered dexamethasone (5 mg/kg) for 5 days prior to LPS injection. The dose of dexamethasone was selected on the basis of previous studies [23,24]

Twenty-four hours after LPS injection, mice were humanely killed under anesthesia using ketamine (50 mg/kg). Right lung was lavaged using 0.9% saline while the left lung was clamped. Bronchoalveolar lavage fluid (BALF) was obtained and centrifuged. Cell pellet was used for the estimation of the cell counts. The supernatants of BALF were stored at −80 °C until further analysis. A small piece of the left lung was weighed, homogenized in phosphate buffer, and centrifuged. The supernatants were stored at −80 °C for further analysis. Another part of the left lung was washed with ice-cold saline and then immersed for 24 h in buffered formalin 10%.

#### 2.7.2. Lung Wet/Dry Weight (W/D) Ratio 

W/D ratio is used to estimate the degree of pulmonary edema. It is calculated as the weight of wet piece of the left lung/its weight after drying in an oven (80 °C) for 24 h [4].

#### 2.7.3. Protein Content 

Samples of BALF were used for estimation of total protein content according to the manufacturer’s kit protocol.

#### 2.7.4. LDH Activity 

The LDH activity was determined in BALF samples on the basis of the protocol of the manufacturer kit. In brief, the reaction mixture consisted of nicotinamide adenine dinucleotide phosphate hydrogen (NADPH) (0.8 mmol/L), and sodium pyruvate (1.5 mmol/L) and Tris buffer (50 mmol/L, pH 7.4) was added to the sample. The changes in absorbance were recorded at 340 nm and enzyme activity was calculated and expressed in U/L.

#### 2.7.5. Total and Differential Cell Counts 

Cell pellets were resuspended in 0.1 mL sterile saline and then centrifuged onto slides and stained with Wright-Giemsa for 8 min. Total cell counts were determined using a hemocytometer. Differential cell counts were quantified by counting a total of 200 cells per slide at 40 × magnification. Number of each cell type was calculated as the percentage of cell type multiplied by the total number of cells in the BALF.

#### 2.7.6. Lung Histology 

Paraffin blocks of lung tissue were obtained from lung samples immersed in formalin, then sectioned (5 µm). Specimens were stained with hematoxylin-eosin (H&E) and examined in random order. Lesions were semi-quantitatively graded as described previously [4].

#### 2.7.7. Immunohistopathology

Immunohistochemistry (IHC) staining was automatically managed using Ventana Benchmark XT system (Ventana Medical Systems, Tucson, AZ, USA). The lung sections were immuno-stained using primary antibodies—rabbit polyclonal antibody to NF-κBp65 following previous procedures [1,25].

#### 2.7.8. Oxidative Stress and Antioxidants 

In the supernatants of lung homogenates, lipid peroxidative markers (MDA and 4-HNE) and antioxidants (catalase, SOD, GSH, and TAC) were determined according to instruction of the manufacturer’s kits.

Briefly, MDA was quantified by the reaction with thiobarbituric acid in acidic medium at a temperature of 95 °C for 30 min to form thiobarbituric acid-reactive product whose absorption was measured spectrophotometrically at 534 nm. Catalase was determined by its reaction with a known quantity of hydrogen peroxide (H_2_O_2_). Catalase inhibitor stopped the reaction after 1 min, and then the remaining H_2_O_2_ reacted with 3,5-dichloro-2-hydroxybenzene sulfonic acid (DHBS) and 4-aminophenazone (AAP) to form a chromophore that was measured at 510 nm. The color intensity was inversely proportional to the amount of catalase in the original sample. Assay of SOD depends on the ability of SOD to inhibit the phenazine methosulphate-mediated reduction of nitroblue tetrazolium dye. The increase in absorbance at 560 nm for 5 min was measured. GSH determination relies on the reaction of GSH with 5,5-dithiobis-2-nitrobenzoic acid. The product was measured spectrophotometrically at 412 nm. The measurement of TAC was performed by the reaction of antioxidants in the sample with a defined amount of H_2_O_2_. The antioxidants in the supernatant interacted with a specific amount of H_2_O_2_. The residual H_2_O_2_ was determined colorimetrically by the conversion of 3,5,dichloro-2-hydroxy benzensulphonate to a colored product that was measured at 510 nm.

#### 2.7.9. NF-κB and Inflammatory Cytokines

Levels of NF-κB, TNF-α, IL-1β, and IL-6 were measured in the supernatants of lung homogenates using ELISA kits. 

#### 2.7.10. Statistical Analysis

Presented results are means ± SD (*n* = 8). Statistical analysis was performed using one-way analysis of variance (ANOVA) followed by Tukey’s Kramer multiple comparisons test. For non-parametric comparison, Kruskal-Wallis test followed by Dunn’s test were used, and a *p*-value < 0.05 was considered significant.

## 3. Results

**Figure 1:**
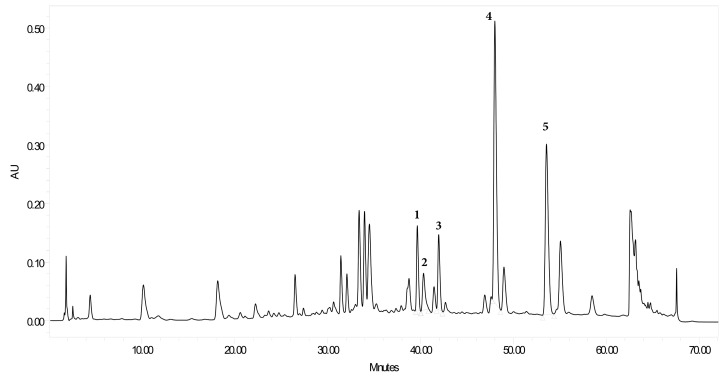
HPLC chromatogram of methanol extract of *Euphorbia cuneata*.

The total methanolic extract (TEC) was standardized for its major phenolic constitutes that were previously isolated [20]. The results showed the presence of 2*R*-naringenin-7-*O*-*β*-glucoside (**1**), kaempferol-7-*O*-*β*-glucoside (**2**), cuneatannin (**3**), quercetin (**4**), and 2*R*-naringenin (**5**) in concentrations of 6.16, 4.8, 51.05, 13.2, and 50 mg/g of extract, respectively (Figure 1).

### 3.1. Effect of EC on LPS-Induced Lung Edema

**Figure 2:**
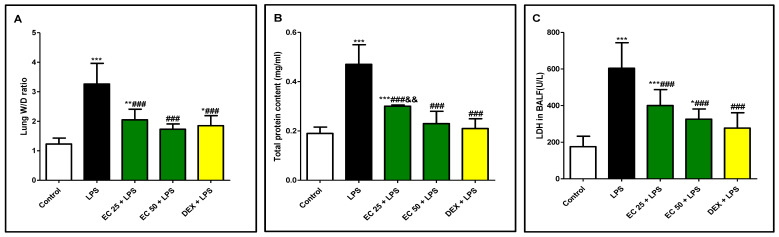
*Euphorbia cuneata* (EC) attenuated lipopolysaccharide (LPS)-induced lung injury. (**A**) Lung wet/dry weight (W/D) ratio, (**B**) protein content, and (**C**) lactate dehydrogenase (LDH) activity in bronchoalveolar lavage fluid (BALF). Mice were administered two different doses of EC (25 and 50 mg/kg) or dexamethasone (5 mg/kg) once daily for 5 days prior to intraperitoneal injection of LPS (10 mg/kg). Samples were collected 24 h after LPS injection. Data are the mean ± SD. (*n* = 8). * *p* < 0.05, ** *p* < 0.01, *** *p* < 0.001 vs. control group; ^###^ *p* < 0.001 vs. LPS group; ^&&^ *p* < 0.01 vs. dexamethasone (DEX) + LPS group (one-way ANOVA).

LPS injection to mice resulted in elevation of lung W/D ratio and total protein content of BALF compared to normal mice, indicating development of pulmonary edema (Figure 2). Additionally, LDH activity was remarkably increased in LPS-treated animals compared to the control group. On the contrary, EC pretreatment as well as dexamethasone significantly attenuated W/D ratio, total protein, and LDH activity in comparison with the untreated LPS group. Interestingly, the effect of EC at a high dose was nearly equivalent to the effect of dexamethasone, as there was no significant difference between EC 50 + LPS group compared to the dexamethasone-treated group (Figure 2).

### 3.2. Effect of EC on LPS-Induced Increase in the Total and Differential Inflammatory Cell Counts in BALF 

**Figure 3:**
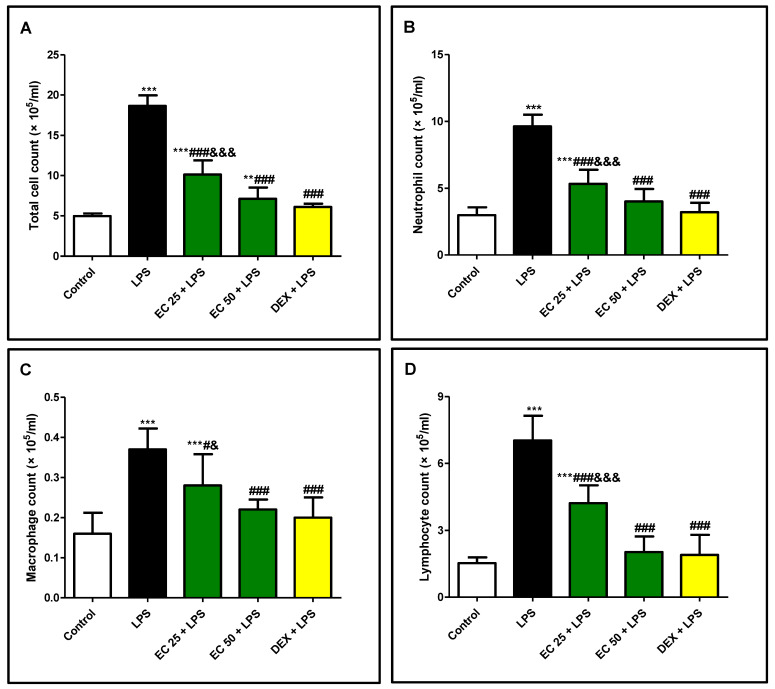
*Euphorbia cuneata* (EC) suppressed lipopolysaccharide (LPS)-induced elevation in total and differential cell counts in bronchoalveolar lavage fluid (BALF). (**A**) Total cell count, (**B**) neutrophil count, (**C**) macrophage count, (**D**) lymphocyte count in lung tissue. Mice were treated with two different doses of EC (25 and 50 mg/kg) or dexamethasone (5 mg/kg) once daily for 5 days prior to intraperitoneal injection of LPS (10 mg/kg). Samples were collected 24 h after LPS injection. Data are the mean ± SD. (*n* = 8). ** *p* < 0.01, *** *p* < 0.001 vs. control group; ^#^ *p* < 0.05, ^###^ *p* < 0.001 vs. LPS group; ^&^ *p* < 0.05, ^&&&^ *p* < 0.001 vs. DEX + LPS group (one-way ANOVA).

As shown in Figure 3, LPS significantly increased the total and differential cell counts, mainly neutrophils, in the BALF compared to the control group. EC or dexamethasone pretreatment significantly suppressed LPS-induced rise in the total and differential cell counts. The effect of EC at a high dose was not significant compared to that of dexamethasone.

### 3.3. Effect of EC on LPS-Induced Lung Damage

**Figure 4:**
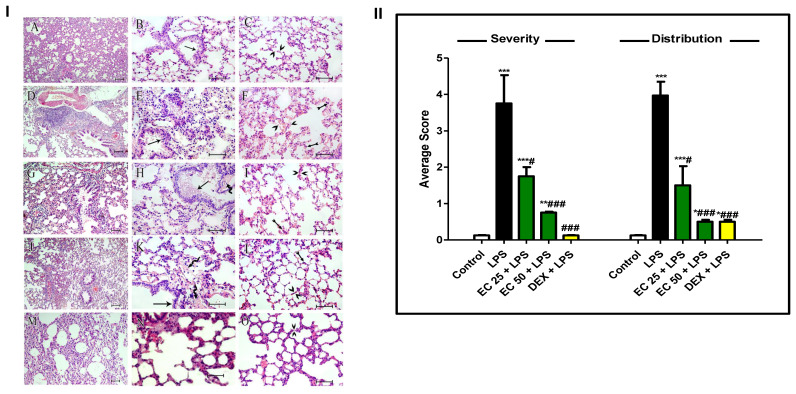
*Euphorbia cuneata* (EC) ameliorated lipopolysaccharide (LPS)-induced histopathological damage of the lung. **I.** Lung specimen of different group stained with hematoxylin-eosin (H&E). (**A**–**C**) **Control group** where lung specimen displayed normal alveolar bronchioles lined by pseudo-stratified ciliated columnar epithelium (arrow), pulmonary blood vessels, interalveolar septae (between arrow heads), alveolar capillaries, and interstitial tissue. (**D**–**F**) **LPS** group showing hypertrophied lining epithelium of the pulmonary bronchiole (arrow) with extravasation of red blood cells (RBCs) and inflammatory cell infiltration in the interalveolar tissue spaces, thickened intralveolar septae (arrow heads) with RBCs extravasation, and extensive neutrophil and macrophage infiltration in the interstitial tissue (tailed arrows). (**G**–**I**) **EC 25 + LPS group,** where the alveolar bronchioles had near normal epithelial lining with interalveolar mucous accumulation (arrow), and lamellae of collagen bundles (curved arrow) is seen close to the bronchiole, with less marked thickened intralveolar septae (arrow heads) with RBC extravasation and scarce neutrophil infiltration in the interstitial tissue (tailed arrow). (**J**–**L**) **EC 50 + LPS group,** where the alveolar bronchioles had near normal epithelial lining without interalveolar mucous (arrow), and lamellae of collagen bundles (curved arrows) are still seen close to the bronchiole, with no RBCs extravasation nor inflammatory cell infiltration in the interstitial tissue and near normal intralveolar septae (arrow heads) with scarce neutrophil infiltration in the interstitial tissue (tailed arrow). (**M**–**O**) **DEX + LPS group,** with near normal intralveolar septae (arrow heads) without neutrophil infiltration nor collagen bundle deposition in the interstitial tissue. **II.** Semi-quantitative analysis of LPS-induced lung lesions with regards to the severity and distribution of the lesions. Mice were administered two different doses of EC (25 and 50 mg/kg) or dexamethasone (5 mg/kg) once daily for 5 days prior to intraperitoneal injection of LPS (10 mg/kg). Samples were collected 24 h after LPS injection. Data are the mean ± SD. (*n* = 8). * *p* < 0.05, ** *p* < 0.01, *** *p* < 0.001 vs. control group; ^#^ *p* < 0.05, ^###^ *p* < 0.001 vs. LPS group (Kruskal-Wallis).

Lung tissue of the control group showed normal histology. There was no sign of lesions in the pulmonary tissue. LPS induced deleterious lung damage in the form of hypertrophied lining epithelium of the pulmonary bronchiole with extravasation of red blood cells (RBCs) and inflammatory cell infiltration in the interalveolar tissue spaces. The thickened inter-alveolar septae were observed with RBC extravasation and excess inflammatory cell infiltration in the interstitial tissue. On the other hand, animals pretreated with EC or dexamethasone exhibited remarkable improvement of the pulmonary lesions compared to the LPS-treated group. Semi-quantitative analysis of LPS-induced lung lesions with regards to the severity and distribution of the lesions indicated significant amelioration of LPS-induced lesions (Figure 4).

### 3.4. Effect of EC on LPS-Induced Lipid Peroxidation and Antioxidants in Lung

**Figure 5:**
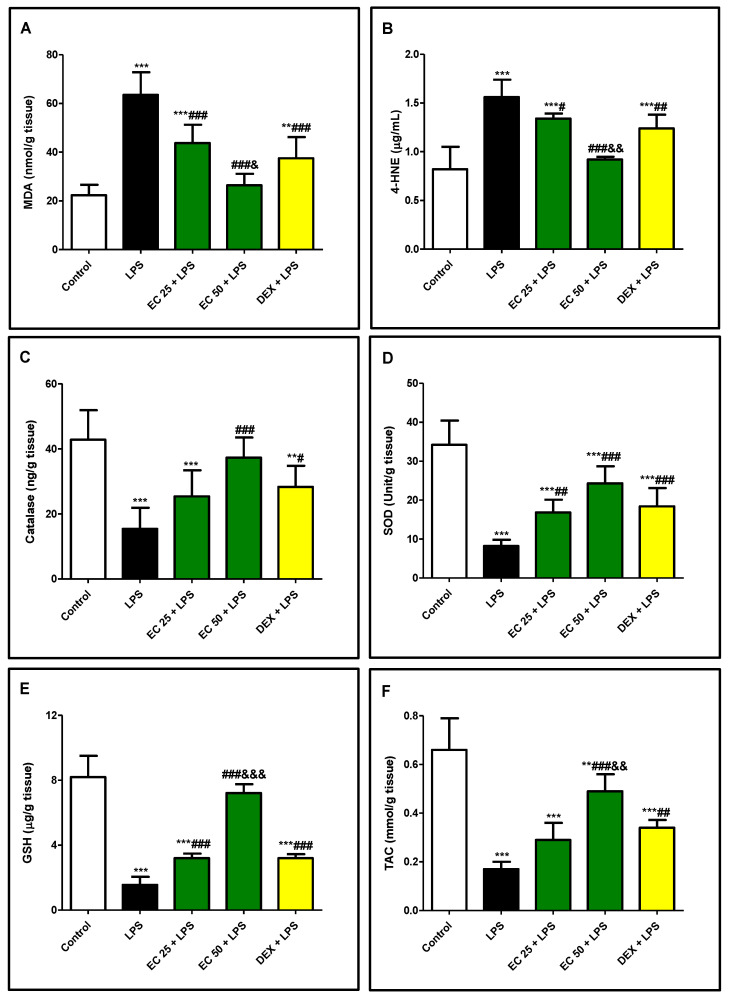
*Euphorbia cuneata* (EC) ameliorated lipopolysaccharide (LPS)-induced lipid peroxidation and increased antioxidant parameters in the lung. (**A**) Malondialdehyde (MDA), (**B**) 4-hydroxynonenal (4-HNE), (**C**) catalase, (**D**) superoxide dismutase (SOD), (**E**) reduced glutathione (GSH), (**F**) total antioxidant capacity (TAC). Mice were treated with two different doses of EC (25 and 50 mg/kg) or dexamethasone (5 mg/kg) once daily for 5 days prior to intraperitoneal injection of LPS (10 mg/kg). Samples were collected 24 h after LPS injection. Parameters were estimated in the supernatants of the lung homogenates. Data are the mean ± SD. (*n* = 8). ** p < 0.01, *** p < 0.001 vs. control group; ^#^ *p* < 0.05, ^##^ *p* < 0.01, ^###^ *p* < 0.001 vs. LPS group; ^&&^ *p* < 0.01, ^&&&^ *p* < 0.001 vs. DEX + LPS group (one-way ANOVA).

LPS injection induced increase in the lipid peroxidative markers, MDA and 4-HNE, in lung in comparison with normal animals (Figure 5A,B). Simultaneously, LPS hindered the antioxidant capacity of the lung due to significant decrease in the endogenous antioxidants such as catalase, SOD, and GSH levels as well as TAC in comparison with the control group (Figure 5C–F). On the other hand, EC or dexamethasone pretreatment significantly augmented the antioxidant activities and diminished the lipid peroxidative parameters in the lung. EC significantly enhanced catalase, SOD, and GSH and significantly decreased MDA and 4-HNE compared to the LPS-treated group. EC at a higher dose exerted a remarkable antioxidant activity compared to dexamethasone.

### 3.5. Effect of EC on LPS-Induced Inflammatory Response in Lung

**Figure 6:**
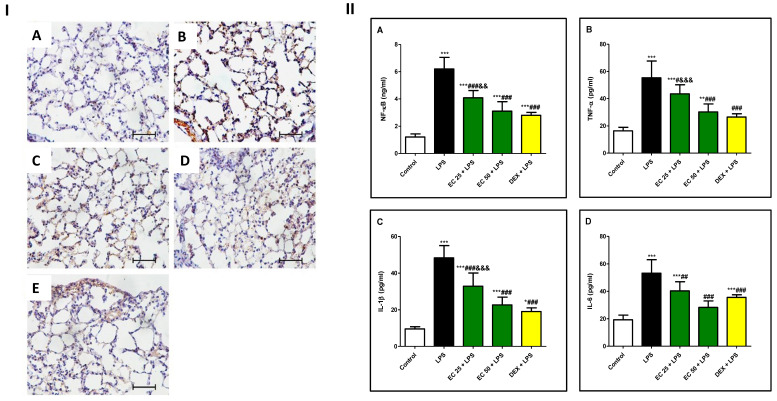
*Euphorbia cuneata* (EC) inhibited lipopolysaccharide (LPS)-induced nuclear factor-κB (NF-κB) activation and cytokine release in lung. **I.** Expression of NF-ĸB cells in lung tissue determined by immunohistochemistry. (**A**) Control group, the positive NF-ĸB cells were not observed; (**B**) LPS group, increased expression of NF-ĸB-positive cells; (**C**) EC 25 + LPS group, there was low staining of NF-ĸB-positive cells; (**D**) EC 50 + LPS group, very limited expression in the perivascular region, and interstitial lung tissue; (**E**) DEX + LPS group, minor positive NF-ĸB cells. **II.** Levels of (**A**) NF-κB, (**B**) tumor necrosis factor-α (TNF-α), (**C**) interleukin-1β (IL-1β), (**D**) interleukin-6 (IL-6) in the supernatants of lung homogenates. Mice were treated with two different doses of EC (25 and 50 mg/kg) or dexamethasone (5 mg/kg) once daily for 5 days prior to intraperitoneal injection of LPS (10 mg/kg). Samples were collected 24 h after LPS injection. Data are the mean ± SD. (*n* = 8). * *p* < 0.05, ** *p* < 0.01, *** *p* < 0.001 vs. control group; ^#^ *p* < 0.05, ^##^ *p* < 0.01, ^###^ *p* < 0.001 vs. LPS group; ^&&^ *p* < 0.01, ^&&&^ *p* < 0.001 vs. DEX + LPS group (one-way ANOVA).

LPS challenge significantly increased the immuno-expression and the level of NF-κB in the lung in comparison with the control group (Figure 6). In addition, LPS elevated the levels of the inflammatory cytokines TNF-α, IL-1β, and IL-6 (Figure 5II) in the lung compared to the control group. However, EC or dexamethasone reduced immuno-expression and the level of NF-κB simultaneously, with significant reduction in the levels of inflammatory parameters TNF-α, IL-1β, and IL-6 in comparison with the LPS group. Notably, the effect of high dose of EC was not significant from the effect of dexamethasone.

## 4. Discussion

ALI is a serious respiratory condition that is characterized by neutrophilia and acute lung inflammation. LPS is an endotoxin derived from Gram-negative bacteria that has been extensively used to establish a model of ALI in rodents. It induces marked pulmonary inflammation after 2–4 h and maximizes at 24–48 h [5]. Thus, in this study, BALF and tissue samples were collected 24 h after LPS exposure. Results of the current study demonstrated the protective antioxidant and anti-inflammatory effects of EC against LPS-induced ALI in mice, which could be related to its ability to modulate the ROS/NF-ĸB/inflammatory cytokine pathway. The anti-inflammatory efficacy of EC, specifically at the higher dose, was nearly equivalent to that of dexamethasone.

LPS administration results in multiple pathogenic events including massive polymorphonuclear leukocytes (PMN) infiltration in pulmonary tissue, diffuse intravascular coagulation, and profound pulmonary injury [6]. Accumulation of infiltered inflammatory cell in pulmonary tissue exacerbates ALI through the release of multiple toxic mediators including ROS, proteases, and proinflammatory cytokines [4]. Furthermore, recruitment of inflammatory cells contributes to increase of the alveolar-capillary barrier permeability and lung edema. Results of this study were in line with previous research [2,10,26], as LPS induced marked pulmonary edema presented by increased lung W/D ratio. The total protein content in the BALF, as another index of epithelial permeability and pulmonary edema [2], was highly increased in the BALF of mice exposed to LPS. However, EC decreased the lung W/D ratio and the total protein in the BALF, indicating that EC could prohibit the leakage of serous fluid into the lung tissue and attenuate the development of pulmonary edema. Additionally, LDH, as a marker of tissue injury, was increased in BALF upon LPS administration compared with the control group, which was inhibited by EC pretreatment. Inflammatory cell infiltration in the lungs, mainly neutrophil, was noted through increased total and differential cell counts. These biochemical observations were supported by the histopathological examination of the lung, which revealed excessive inflammatory changes such as pulmonary edema, alveolar distortion, and inflammatory cell infiltration lung of LPS-challenged mice. On the other hand, EC attenuated pulmonary edema and inhibited the infiltration of inflammatory cells, as well as restraining the alveolar structural damage. The remarkable attenuation of the biochemical parameters of ALI was parallel to the observed improvement of the histology of the lung in EC-pretreated animals. These findings demonstrated the protective effect of EC on ALI induced by LPS and the ability of EC to prohibit inflammatory cell sequestration and migration into the lung tissue.

Multiple molecular mechanisms mediate the pathogenic events of LPS-induced ALI [27]. Interaction between oxidative stress and inflammation plays a major role in mediating LPS-induced ALI [28]. During the inflammatory response, neutrophils undergo a respiratory burst and produce superoxide. ROS overproduction is extremely toxic to host tissues, and their interactions with various cellular macromolecules result in severe pathophysiological consequences. Excessive LPS-induced ROS release is accompanied by production of lipid peroxides, inactivation of proteins, and DNA mutation [5]. Moreover, LPS-induced oxidative stress is associated with depressed antioxidant activity of the lung, which may aggravate LPS toxicity. Catalase is one of the most important antioxidant enzymes that antagonizes oxidative stress by destroying cellular hydrogen peroxide to produce water and oxygen. GSH acts as a major cellular antioxidant defense system by scavenging free radicals and other ROS. SOD is the only antioxidant enzyme that can scavenge superoxide. Catalase, GSH, and SOD are greatly depressed by LPS [1,2,5]. Our results are consistent with previous studies, as LPS caused marked lipid peroxidation. LPS increased MDA and 4-HNE, which are stable end-products of lipid peroxidation and are frequently used as biomarkers of oxidative stress. Moreover, LPS repressed the activities of the antioxidants (catalase, SOD, GSH, TAC) in lung tissue. Significantly, EC reversed LPS-induced oxidative changes as EC enhanced antioxidant activities and subsequently depressed lipid peroxidation, clearly demonstrating the potent antioxidant activity of EC.

LPS binds and stimulates toll-like receptor 4 (TLR4), which causes activation of NF-κB and subsequent release of inflammatory cytokines (TNFα, IL-1β, and IL-6) [29,30]. Hence, blockade of NF-κB signaling pathways can inhibit the development of ALI induced by LPS. Our study revealed the activation of NF-κB and increased inflammatory cytokines in LPS-challenged mice, consistent with previous studies [4]. Expectedly, pretreatment with EC hindered the activation of this inflammatory pathway, resulting in suppression of inflammatory mediator release. It is worth mentioning that jolkinolides, diterpenoids reported from *Euphorbia* species, have shown a protective effect of LPS-induced ALI via attenuating histological alterations, inflammatory cell infiltration, and lung edema, as well as inhibiting the production of inflammatory mediators, e.g., TNF-α [31,32]. It has also been reported that *Euphorbia* factor L2 improved the survival rate of ALI mice and effectively reduced the pathological changes in the lung by suppression of pro-inflammatory mediators regulated by the NF-κB pathway [33]. As noted above, EC contains several phytochemicals, including quercetin, kaempferol glycoside, and naringenin. Quercetin is known for its antioxidant and anti-inflammatory activity through inhibition of TNF-α, IL-8, IL-4, cyclooxygenase (COX), and lipoxygenase (LOX) [34]. Naringenin has been shown to exhibit anti-inflammatory activity in lung injury in vivo through downregulation of NF-κB, inducing NO synthase, TNF-α, and caspase-3 [35]. Kaempferol glycoside, which is biosynthesized from naringenin, is known for its antioxidant potential, in addition to its anti-inflammatory effect through inhibition of NF-κB, TNF-α, COX, LOX, and expression of IL-1β and IL-8 [36]. Thus, the protective activity of EC against LPS-induced ALI in mice may be attributed to its antioxidant activity due to the presences of the different phenolic constituents.

## 5. Conclusions

Collectively, this study revealed the potent protective effect of EC against LPS-induced ALI, which may be linked to its antioxidant and anti-inflammatory activities. However, further studies are recommended for better elucidation of the underlying molecular mechanisms of EC.
